# Aerosol and Contact Transmission Following Intranasal Infection of Mice with Japanese Encephalitis Virus

**DOI:** 10.3390/v11010087

**Published:** 2019-01-21

**Authors:** Chunxia Chai, Rachel Palinski, Yixuan Xu, Qiao Wang, Sanjie Cao, Yi Geng, Qin Zhao, Yiping Wen, Xiaobo Huang, Qiguai Yan, Xiaoping Ma, Xintian Wen, Yong Huang, Xinfeng Han, Wenjun Ma, Rui Wu

**Affiliations:** 1 Research Center of Swine Disease, College of Veterinary Medicine, Sichuan Agricultural University, Chengdu 611130, China; chaichunxia_ccx@126.com (C.C.); xyxtk@foxmail.com (Y.X.); wangqiao666666@126.com (Q.W.); csanjie@sicau.edu.cn (S.C.); zhaoqinde@foxmail.com (Q.Z.); yueliang5189@163.com (Y.W.); rsghb110@126.com (X.H.); yanqigui@126.com (Q.Y.); mxp886@sina.com.cn (X.M.); Xintian3211@126.com (X.W.); hyong601@163.com (Y.H.); hanxinf@163.com (X.H.); 2 Department of Diagnostic Medicine/Pathobiology, College of Veterinary Medicine, Kansas State University, Manhattan, KS 66506, USA; rpalinski@vet.k-state.edu; 3 College of Veterinary Medicine, Sichuan Agricultural University, Chengdu 611130, China; gengyisicau@126.com

**Keywords:** JEV, contact transmission, aerosol transmission, intranasal infection, mice

## Abstract

The Japanese encephalitis virus (JEV), a causative agent of severe viral encephalitis in humans, has a biological cycle fluctuating between transmission in mosquitoes and avian species and amplification in pigs. Contact transmission of JEV was recently shown in pigs in the absence of arthropod vectors. Here, we show JEV transmission between infected and contact mice and further demonstrate that JEV transmission occurs between animals via aerosols, as both viral RNA and infectious JEV were detected in direct contact- and aerosol-exposed contact animals. The results of this study change our understanding of JEV transmission in densely populated regions and may help to explain JEV outbreaks without the presence of arthropod vectors.

## 1. Introduction

Japanese encephalitis (JE) is a zoonotic vector-borne viral disease, causing the encephalitis most frequently associated with fatal or severe outcomes in humans [1]. It is currently present in Asia and Australia with 50,000–175,000 cases occurring annually [2,3]. Human cases generally occur in rural areas or at the edges of cities following heavy rains or monsoons [1]. In these cases, less than 1% of patients develop encephalitis; however, 20–30% of encephalitic patients succumb to the disease. Most notably, ~50% of patients surviving encephalitis suffer neurologic or psychologic sequelae [4]. 

Japanese encephalitis virus (JEV), the causative agent of JE, is a mosquito-borne member of the genus Flavivirus, family *Flaviviridae*. *Culex* mosquitoes are the main vector for JEV, while wild water birds represent the reservoirs of the virus [5]. Swine are highly susceptible to JEV infection, although the clinical signs generally remain subclinical, and this species serves as amplifying hosts during human epidemics [5,6,7,8]. Neurotropic disease occurs relatively rarely in the virus’ natural hosts, although abortion has been noted in pregnant sows [1]. In contrast, humans and horses are considered dead-end hosts, as they may develop fatal disease but do not develop a viremia capable of transmitting the virus.

Despite the previously established vector–host cycle of JEV, no virus was isolated from locally collected mosquitoes during or prior to recent epidemics [9,10]. Furthermore, transfusion-related JEV transmission has been reported in patients in Hong Kong [11]. Together, these facts suggest that JEV is capable of vector-free transmission. 

In 2016, the vector-free transmission and viral persistence of JEV was experimentally confirmed in pigs. Pigs were not only susceptible to oronasal infection, but the virus was shown to persist in the tonsils for 25 days despite the detection of high titers of neutralizing antibodies. JEV RNA was detected in oral fluids for up to 14 days following infection in swine, and it seems to be the route of viral exit and entry for pigs [12,13]. The high susceptibility of infection coupled with a low infectious dose suggests vector-free transmission is important to natural infection cycles and prompts the examination of other routes of vector-free transmission [14].

In this study, we examine the capabilities of two different genotypes of JEVs, which also differ in virulence, to transmit in mice via either contact or aerosol routes. The designed studies aim to determine the potential of viral shedding and transmission to better understand non-vector-borne disease outbreaks. First, we define the tissue tropism of both viruses when administered via the intranasal route, then we determine the dose response to JEV infection in the mouse lung and the potential of contact transmission of JEV in mice. Finally, we determine the potential of aerosol transmission among mice.

## 2. Materials and Methods

### 2.1. Ethics Statement

All the animal experiments were carried out in strict accordance with the recommendations in the Guide for the Care and Use of Laboratory Animals of the Ministry of Science and Technology of the People’s Republic of China. The protocols in this study were approved by the Institutional Animal Care and Use Committee of Sichuan Agricultural University (IACUC#RW2016-090).

### 2.2. Viruses, Cells, and Animals 

The SCYA201201 strain is a genotype I JEV that is neurovirulent in mice and was isolated from diseased pigs in China in 2012 [15]. The SA14-14-2 strain is a widely used live, attenuated JEV vaccine strain, which is a genotype III lineage JEV that is non-neurotropic [16,17]. Baby hamster kidney (BHK-21) cells were maintained as described previously [18]. 

In addition, 4-week-old female specific pathogen-free (SPF) BALB/c mice were purchased from Chengdu Dossy Experimental Animal Co., Ltd. (Chengdu, China) and housed under Biosafety Laboratory 2 (BSL-2) conditions. The mice were allowed to acclimate for 3 days prior to infection, and their clinical signs were monitored daily throughout the study. 

### 2.3. Mouse Experiments

A total of 5 mouse studies were performed to determine JEV tissue tropism, dose responses, the possibility of contact transmission, and the potential for aerosol transmission. 

To evaluate JEV tissue tropism, 2 groups of 15 mice were inoculated intranasally (i.n.) with 10^6.0^ plaque-forming units (pfu)/50 μL of either the SCYA201201 or SA14-14-2 virus. A third group of 15 control mice were administered equal volumes and inoculation routes of phosphate-buffered saline (PBS). On 3, 4, 5, 6, and 7 days post-inoculation (dpi), 3 mice from each group were humanely euthanized, and their lungs, tracheas, brains, thymuses, livers, spleens, and kidneys were collected for molecular analysis. 

In the second study, to test dose responses in mice, 6 groups of 15 mice were inoculated i.n. with 1 of 3 different doses (10^2.0^, 10^4.0^, or 10^6.0^ pfu/50 μL/mouse) of either the SCYA201201 or SA14-14-2 virus. Then, 1 group of 15 control mice was inoculated with equal volumes of PBS. At 3, 5 and 7 dpi, oronasal swabs were collected, and the viral RNA load was determined using real-time RT-PCR (qRT-PCR). Next, 3 animals in each group were humanely euthanized on 3, 5, 7, 11, and 15 dpi. Serum, lung, and brain samples were collected for further analyses. The lung samples were homogenized for qRT-PCR detection, while the serum samples were tested for the presence of neutralizing antibodies via the plaque reduction neutralization test (PRNT). The lung and brain samples taken at 7 dpi were used for histopathological analyses. In the third mouse study, 2 groups of 21 mice were i.n. inoculated with 10^6.0^ pfu/50 μL of either the SCYA201201 or SA14-14-2 virus. Then, 1 group of 21 control mice was inoculated with equal volumes of PBS. Next, 3 animals in each group were humanely euthanized on 3, 4, 5, 6, 7, 11, and 15 dpi, and their lungs were collected at each necropsy date to determine the viral RNA load using qRT-PCR.

To evaluate the possibility of contact transmission in the mice, two groups of 9 mice were i.n. inoculated with 10^6.0^ pfu/50 μL of either the SCYA201201 or SA14-14-2 virus, respectively. Then, 1 group of 9 mice was housed separately and i.n. inoculated with PBS as the controls. After 24 h post-infection, 9 sentinel mice were placed in each cage. On 3, 5, and 7 dpi, 3 infected and 3 sentinel mice were humanely euthanized from each group, and lung samples were collected for molecular analyses and virus isolation. The serum samples were tested for the presence of neutralizing antibodies by PRNT. The lung samples were homogenized for qRT-PCR or serially diluted in Dulbecco’s modified Eagle medium (DMEM) for viral titration. 

To determine the potential for aerosol transmission in mice, 2 groups of 9 animals were i.n. inoculated with 10^6.0^ pfu/50 μL of either the SCYA201201 or SA14-14-2 virus, respectively. Then, 9 mice were i.n. inoculated with PBS and housed separately as the controls. The animals were housed on one side of a cage with a double-walled wired partition that allowed airflow but prevented bedding, feces, and urine contamination, as well as contact with the other side. After 24 h post-inoculation, 9 sentinel animals were added to the empty side of the cage. On 3, 5, and 7 dpi, 3 animals from the infected groups and 3 animals from the sentinel groups were humanely euthanized, and lung samples were collected for further analyses. The serum samples were tested for the presence of neutralizing antibodies by PRNT.

### 2.4. Real-time RT-PCR for the Detection of JEV

The qPCR was performed as described previously using specific primers targeting the JEV envelope [19,20]. 

### 2.5. Neutralizing Antibody Quantification Using the Plaque Reduction Neutralization Test

After infection, orbital blood was collected from the mice on the indicated days. To determine the neutralizing antibody titers of the serum samples, the reciprocal of the maximum dilution of serum that yielded a 50% plaque reduction (PRNT_50_) was calculated as described previously [21,22].

## 3. Results

### 3.1. Tissue Tropism of Intranasal Inoculation of JEV in Mice

**Figure 1:**
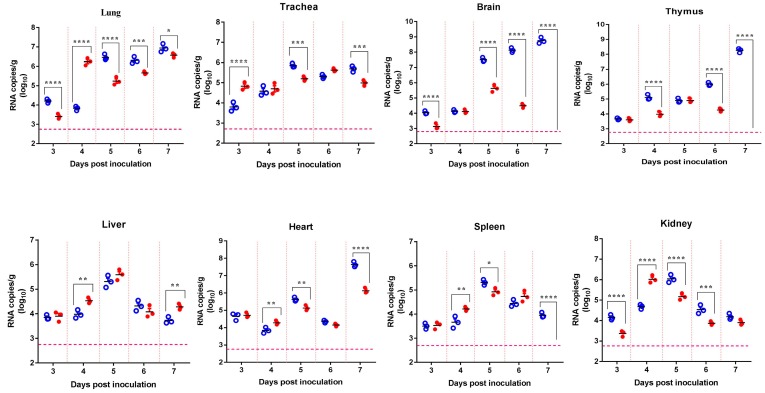
Tissue distribution of Japanese encephalitis virus (JEV) RNA in mice infected by the intranasal route. Four-week-old mice were intranasally (i.n.) inoculated with 10^6.0^ plaque-forming units (pfu)/50 μL of SCYA201201 or SA14-14-2 JEV virus. The viral RNA load was measured using real-time RT-PCR in each mouse tissue collected at 3, 4, 5, 6, and 7 days post-infection (dpi) and expressed as RNA copies per gram (Mean ± SEM) (○ represent the SCYA201201 strain, and ● represent the SA14-14-2 strain). The horizontal dotted lines indicate the limit of detection (* *P* ˂ 0.05, ** *P* ˂ 0.01, *** *P* ˂ 0.001, and **** *P* ˂ 0.0001).

A total of two groups of 15 mice were i.n. inoculated with either SCYA201201 or SA14-14-2 and sequentially sacrificed on 3, 4, 5, 6, and 7 dpi to evaluate the viral tissue tropism. No obvious clinical signs such as weight loss, neurological signs, or depression were observed in any of the infected or control mice during the experiment. Viral RNA was detected in all the tested tissues, including the lungs, tracheas, brains, thymuses, livers, hearts, spleens, and kidneys of the infected mice with either virus (Figure 1). As expected, SCYA201201 replicated more efficiently than SA14-14-2, especially at the later time points from 5–7 dpi in all the tissues except in the liver. Although both viruses replicated efficiently in the lungs, the level of viral RNA detected in the SCYA201201-infected mice was significantly higher than that found in the SA14-14-2-infected animals at 3, 5, 6, and 7 dpi (Figure 1). In contrast, a significantly higher RNA load was observed in the lungs of the SA14-14-2-infected mice at 4 dpi. The results of this study indicate that both JEV strains cause systemic infection in mice inoculated by the intranasal route and replicate to a greater extent in the lung. 

### 3.2. Intranasal Inoculation Dose Response in the Lungs of Mice

**Figure 2:**
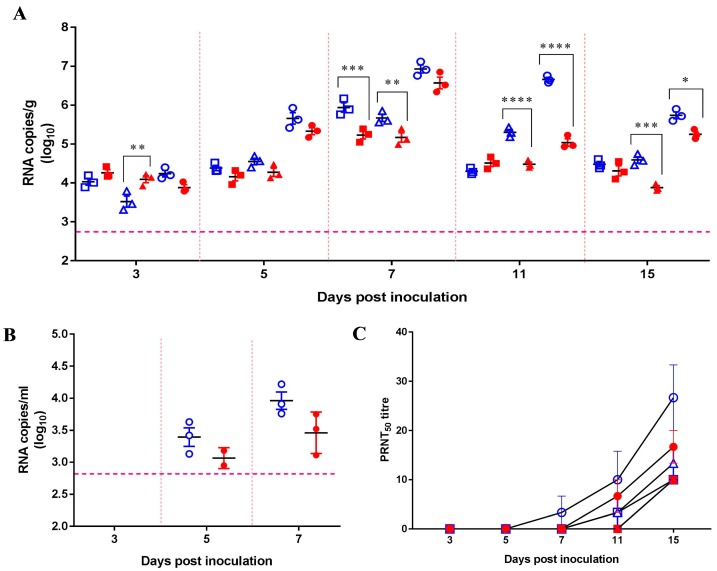
Virus replication, shedding, and seroconversion in mice intranasally inoculated with JEV. Four-week-old mice were i.n. inoculated with different doses (10^2^, 10^4^, or 10^6^ pfu/50 μL) of the SCYA201201 or SA14-14-2 virus. The SCYA201201 virus is marked with hollow symbols (10^2.0^ with □, 10^4.0^ with △, and 10^6.0^ with ○), while the SA14-14-2 virus is marked with solid symbols (10^2.0^ with ■, 10^4^ with ▲, and 10^6^ with ●). (**A**) The viral RNA load in lungs at 3, 5, 7, 11, and 15 dpi is measured using real-time RT-PCR and expressed as RNA copies per gram (Mean ± SEM). (**B**) The viral RNA load in oronasal swabs collected at 3, 5, and 7 dpi was measured using real-time RT-PCR assay and expressed as RNA copies per mL (Mean ± SEM). (**C**) The sera were collected from infected or uninfected mice at 3, 5, 7, 11, and 15 dpi, and neutralizing antibodies were detected by PRNT. The horizontal dotted lines indicate the limit of detection (*P ˂ 0.05, ** *P* ˂ 0.01, *** *P* ˂ 0.001, and **** *P* ˂ 0.0001)).

As both JEVs replicated efficiently in mouse lungs after i.n. inoculation with a high dose (10^6^ pfu) of JEV, we further examined the viral replication in the lungs and viral shedding using different doses of the viruses. A total of six groups of 15 mice were i.n. inoculated with 10^6^, 10^4^, or 10^2^ pfu/50 μL of either the SCYA201201 or SA14-14-2 virus. No obvious clinical signs, including weight loss or neurological signs, were observed in any of the experimental mice throughout the length of the study. In the 10^2^ pfu/50 μL-infected mice, the viral RNA was not significantly different between the groups at all the tested dates except for 7 dpi, at which point a significantly higher amount of viral RNA was found in the SCYA201201-infected mice. In contrast, the 10^4^ and 10^6^ pfu/50 μL SCYA201201 groups displayed significantly higher levels of viral RNA at 11 and 15 dpi when compared with the SA14-14-2-infected animals (Figure 2A). The viral RNA load was determined from oronasal swabs collected from mice infected with 10^6^ pfu/50 μL of both viruses at 3, 5, and 7 dpi. The results showed no statistical differences between the two viruses, although a higher viral RNA load was found in the SCYA201201-infected mice at both 5 and 7 dpi (Figure 2B). 

No significant difference in the viral RNA load was detected in the lungs of the SCYA201201-infected mice at 3, 5, and 7 dpi when compared to those inoculated with the SA14-14-2 virus (Figure 2A). These results are not in agreement with those obtained in the first mouse study (Figure 1), in which the mice were inoculated similarly with 10^6.0^ pfu/50 μL of each virus. To confirm the results in the first two mouse studies, two groups of 21 mice were i.n. inoculated with 10^6.0^ pfu/50 μL of each virus. As expected, the viral RNA load in the mouse lungs was comparable to the two aforementioned experiments. Furthermore, a significantly higher viral RNA load was found in the lungs of the mice inoculated with the SCYA201201 virus at 3, 6, 7, 11, and 15 dpi but not at 4 and 5 dpi. The observed differences are most likely due to the variation in inoculation efficiency. Noticeably, high viral RNA loads in both 10^6.0^ pfu/50 μL of the SCYA201201 and SA14-14-2 infection groups lasted through 15 dpi, indicating that JEV can sustainably replicate in the lungs of mice.

The appearance of neutralizing antibodies was assessed by PRNT in serum samples collected in the second mouse study at 3, 5, 7, 11, and 15 dpi. A neutralization antibody titer was only detected in a single mouse on 7 dpi in the 10^6.0^ pfu/50 μL SCYA201201 inoculation group. The mice in the remaining groups displayed a neutralization antibody titer (≥1:10) at 11 or 15 dpi (Figure 2C). All the animals in the SCYA201201 groups had a higher antibody titer when compared with the SA14-14-2 groups infected with the same dose. Noticeably, at least a 1:20 titer was observed in the mice infected with 10^6.0^ pfu/50 μL of SCYA201201 at 15 dpi. In contrast, the mice infected with the same dose of the SA14-14-2 virus displayed 1:10 or 1:20 antibody titers on the same day (Figure 2C). The molecular and serological data together indicate that JEV can infect and replicate in the mouse lungs with the potential to transmit via the oronasal route.

**Figure 3:**
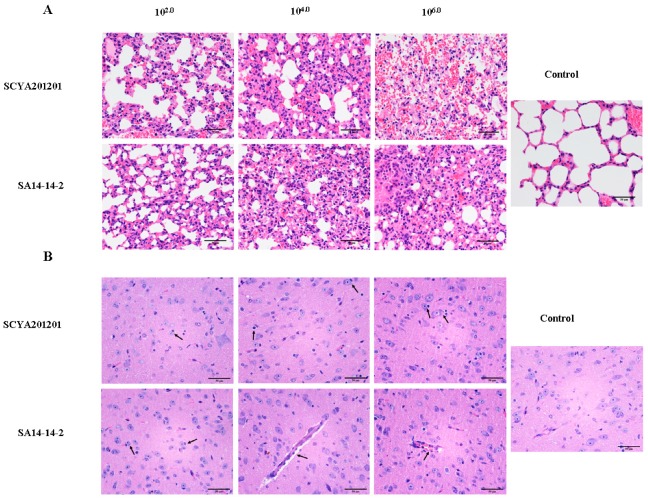
Hematoxylin and eosin (H+E) analysis of mouse lung and brain sections. Four-week-old mice were i.n. inoculated with different doses (10^2^, 10^4^, or 10^6^ pfu/50 μL) of the SCYA201201 or SA14-14-2 virus. (**A**) H+E staining of mouse lung sections revealed pathologic changes consistent with JEV infection. The scale indicates 50 μm. (**B**) The H+E staining of mouse brain sections revealed brain lesions consistent with JEV infection. The brain lesions are indicated by arrows. There are no microscopic lesions in the lung and brain of the control mice. The scale indicates 50 μm.

To determine the pathology, the lungs and brains of the mice infected with SCYA201201 or SA14-14-2 at 10^2.0^, 10^4.0^, or 10^6.0^ pfu/50 μL JEV were harvested at 7 dpi. Compared with the lungs of the control mice, pathologic lesions were seen in the lungs of the mice infected with both viruses by hematoxylin and eosin (H+E) staining. In both groups, the macrophages were present in the alveoli, and the alveolar wall appeared thickened. In addition, severe alveolar hemorrhage was present in the mice infected with 10^6.0^ pfu/50 μL of the SCYA201201. No microscopic lesions were seen in the alveoli of the control mice (Figure 3A). In the H+E-stained brain sections, pathologic lesions were seen in the mice infected with both viruses at 7 dpi. The brain lesions were characterized by apyogenous encephalitis, including increased glial cells, neuronophagia, neuronal degeneration and necrosis, and perivascular lymphocytic infiltration. There were no microscopic lesions in the brain of the control mice (Figure 3B). No significant differences were observed in the brain lesions induced by both viruses with different inoculation doses.

### 3.3. Contact Transmission of JEV in Mice

**Figure 4:**
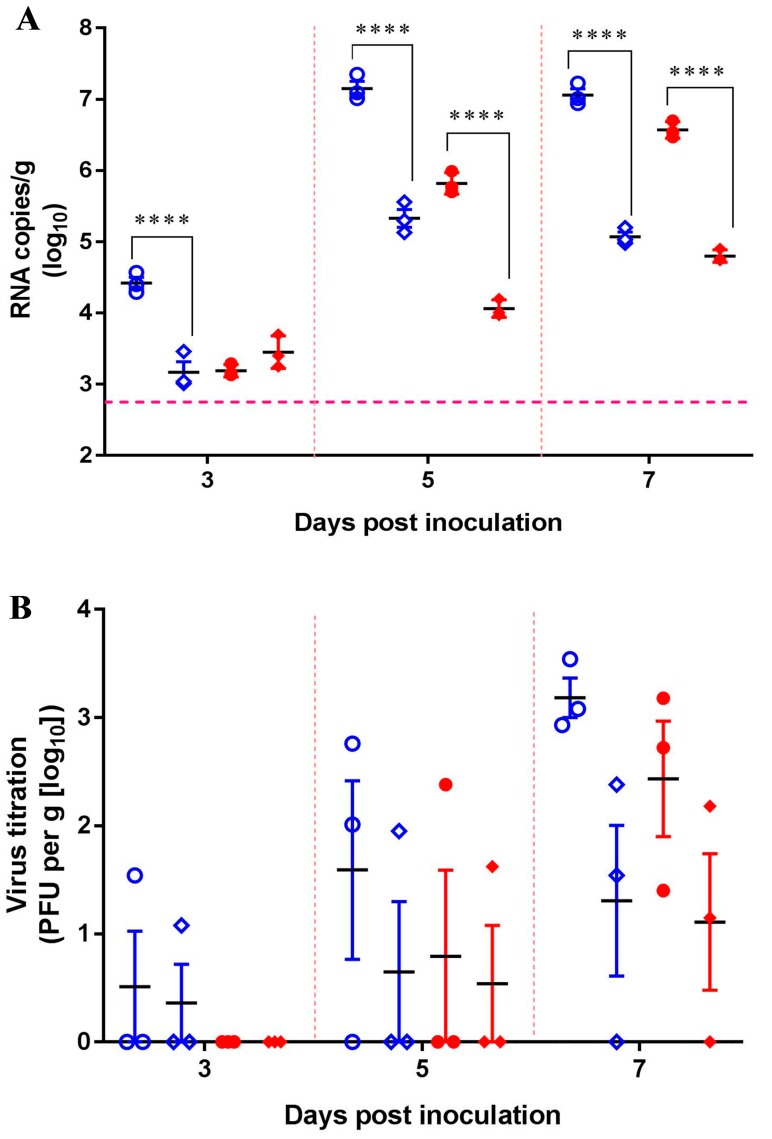
Contact transmission of JEV in mice. Mice were i.n. infected with 10^6.0^ pfu/50 μL of the SCYA201201 or SA14-14-2 virus. The sentinel mice were commingled with the infected animals at 24 h post-infection. The SCYA201201 strain is marked with hollow symbols (infected with ○ and sentinel with ◇), while the SA14-14-2 strain is marked with solid symbols (infected with ● and sentinel with ◆). (**A**) The viral RNA load in the mice lungs harvested at 3, 5, and 7 dpi was measured using real-time RT-PCR. The horizontal dotted lines indicate the limit of detection (**** *P* ˂ 0.0001). (**B**) The virus titer in the lung samples was determined by plaque assay and was presented using plaque-forming units (pfu) per gram of lung tissue.

Two groups of nine mice were i.n. inoculated with 10^6.0^ pfu/50 μL with either SCYA201201 or SA14-14-2 and housed separately. At 24 h post-infection, nine sentinel mice were placed in each infected group to assess the contact transmission of JEV. Based on our previous results, in which JEV replicated efficiently in mouse lungs and was detected in oronasal swabs of infected mice, we focused on determining the viral loads in the mouse lungs in subsequent experiments. Viral RNA was detected in the lungs of all the contact and infected mice with both viruses at all the tested dates, although to a lesser extent at 3 dpi (Figure 4A). At 5 and 7 dpi, both JEV strains showed significantly higher viral RNA loads in the infected mice than those detected in the sentinel animals by 1–2 orders of magnitude. At the termination of the study on 7 dpi, the sentinel animals showed viral loads of 10^5.07^ and 10^4.80^ RNA copies/g in the lungs of the SCYA201201 and SA14-14-2-infected groups, respectively (Figure 4A). The presence of the infectious virus in the lungs of all the infected and sentinel animals was confirmed by the plaque assay, with titers of 10^1.0^–10^3.0^ pfu/g on 3, 5, and 7 dpi (Figure 4B). Noticeably, SCYA201201 was detected in both the infected and contact mice at 3, 5, and 7 dpi in contrast with SA14-14-2, which was only found at 5 and 7 dpi (Figure 4B). However, a neutralization antibody was not detected in all the serum samples of all the infected and sentinel mice on 3, 5, and 7 dpi (data not shown). This result confirms that the SCYA201201 virus replicates and transmits more efficiently in mice than the SA14-14-2 virus. Taken together, these data indicate the contact transmission of JEV to naïve animals.

### 3.4. Aerosol Transmission of JEV in Mice 

**Figure 5:**
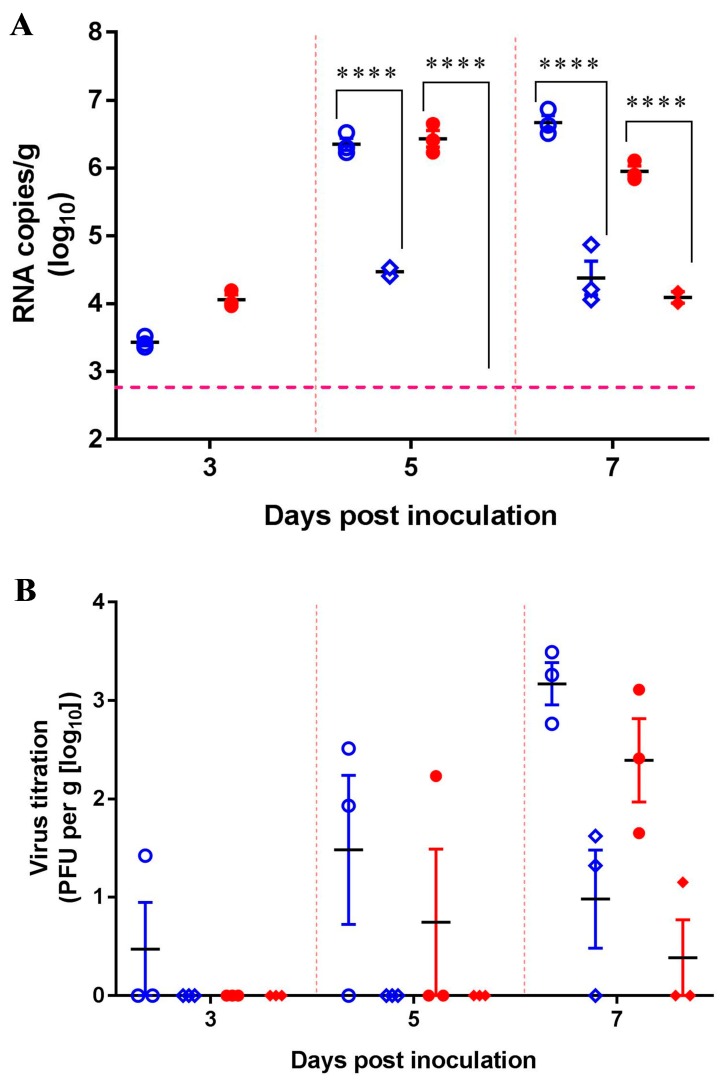
Aerosol transmission of JEV in mice. The mice were infected i.n. with 10^6.0^ pfu/50 μL of the SCYA201201 or SA14-14-2 virus, respectively, and were housed in a cage with a double-walled wire partition that permitted airflow but prevented direct contact. The sentinel animals were added at 24 h post-infection. The mice were sacrificed, and their lungs were harvested at 3, 5, and 7 dpi. The SCYA201201 was marked with hollow symbols (infected with ○ and sentinel with ◇) and the SA14-14-2 was marked with solid symbols (infected with ● and sentinel with ◆). (**A**) The viral RNA load in the lungs was measured using real-time RT-PCR assay. The horizontal dotted lines indicate the limit of detection (**** *P* ˂ 0.0001). (**B**) The virus titer in the lung samples were determined by the plaque assay.

Two groups of nine mice were i.n. inoculated with 10^6.0^ pfu/50 μL of either SCYA201201 or SA14-14-2. An equal number of sentinel mice were housed separately. The infected and sentinel mice were separated by a double-walled mesh wire, which prevented direct contact but facilitated aerosol transmission. As expected, all the animals in the infected groups displayed detectable viral RNA loads in the lungs at 3, 5, and 7 dpi (Figure 5A). The viral load of the infected animals peaked at 7 dpi at 10^6.67^ and 10^6.43^ RNA copies/g for SCYA201201 and SA14-14-2, respectively. None of the sentinel animals displayed detectable viral RNA loads at 3 dpi, but two out of three SCYA201201-infected mouse lungs displayed 10^2.8^ RNA copies/g at 5 dpi. At 7 dpi, viral RNA was detected in the lungs of three out of three or two out of three mice with an averaged titer of 10^4.38^ and 10^2.73^ RNA copies/g in the SCYA201201 and SA14-14-2 groups, respectively (Figure 5A). A higher titer of infectious virus was found in the lungs of the SCYA201201-infected mice on 3, 5, and 7 dpi when titrated by the plaque assay. In contrast, SA14-14-2 was only found at 5 and 7 dpi. Infectious viruses were detected at 7 dpi in the sentinel mice of both virus infection groups (Figure 5B). However, a neutralization antibody was not detected in all the serum samples of the infected and sentinel mice on 3, 5, and 7 dpi. Our results indicate that JEVs are capable of aerosol transmission in mice under experimental conditions. 

## 4. Discussion

JEV has been thought to transmit solely through mosquito vectors. The *Culex* species has been implicated as the main contributors to the spread of the disease. The concept that mosquitoes are the sole contributors to JEV outbreaks and disease has been questioned recently because epidemiological and virologic data on outbreaks in non-tropical regions suggests that other factors also play a role in the spread of the disease. In 2016, Ricklin et al. proved the contact transmission of JEV in experimentally infected pigs; the results of the study suggest contact transmission plays a more important role in viral dissemination in large, dense populations of animals [14]. Utilizing mice in this study is advantageous, as human and mouse JEV infections result in similar physiopathological markers and clinical signs of disease [23,24]. In this study, we not only confirm that contact transmission is possible but also demonstrate that aerosol transmission may occur in the mouse model. The results of these studies add to our understanding of JEV transmission within populations of susceptible species without the presence of mosquito vectors. 

Confirming previous findings, our results indicate that mice are highly susceptible to certain strains of JEV. Even a low viral dose of 10^2.0^ pfu/50 μL caused a sustained infection in mice when i.n. inoculated with either the SA14-14-2 or SCYA201201 JEV strain. The vaccine strain SA14-14-2 was generated by attenuating the parent strain SA14 in suckling mice and has been shown to replicate efficiently in mice infected with as low as 1.5 pfu/mouse [16,17]. The virulent SCYA201201 was isolated in 2012 from aborted pigs in Sichuan province, China. The virus was found to replicate in mice, but the dose response was not determined [15]. Interestingly, the low dose of both JEV strains resulted in similar RNA loads in the lungs through 15 dpi; however, both high dose groups showed significant differences in the viral RNA load at the later timepoints of 11 and 15 dpi. It is unclear whether the replication observed in the lungs is local replication due to the infection method or a possible site of JEV persistence. In contrast to our former mouse studies, in which 7-day-old suckling mice were subcutaneously inoculated with the SCYA201201 virus, no virus was detected in the lungs of the infected mice that displayed neurological signs and died between 5 and 7 dpi [13]. The same study also showed that SCYA201201 subcutaneous inoculation resulted in disease and mortality in 3-week-old mice [13]. In the present study, no mice showed clinical signs or mortality after intranasal inoculation with the virulent SCYA201201 virus, although a high viral load was detected in both the lungs and brains. The differing results could be explained by a difference in the infection routes, where intranasal inoculation causes brain infection via the olfactory nerve and subcutaneous inoculation does not. JEV has been found to persist in the tonsils after oronasal infection in pigs, although the infection dynamics and clinical presentation in dead-end hosts, such as humans and mice, differ from that of amplifying hosts, such as pigs [1,14]. The virus replication in the lungs were not examined in the study; however, the persistence of JEV may explain the epidemiology of past outbreaks. Future studies are necessary to determine the viral replication and persistence in different routes of infection, as well as the disease course and prognosis. 

The pathology of the infected groups is consistent with the previous findings for JEV and other encephalitic flaviviruses [15,25,26,27]. In mice i.n. infected with different doses of the virus, lung and brain lesions were observed in the mice infected with both viruses. Alveolar hemorrhage in the lungs and neuronal necrosis in the brain were also observed in mice infected intracerebrally with JEV [27]. Neuronal degradation has occurred in the brain of mice infected with West Nile virus, or Zika virus as well [25,26]. Interestingly, no obvious difference in the brain lesions was observed between the virulent and attenuated vaccine strains of JEVs used, which is most likely due to the intranasal infection route used in this study, because no mortality was found in the mice infected with a high dose of the virulent virus. Overall, the pathology observed in mice in our study was caused by JEV infection. 

The replication of JEV in mouse lungs in this study suggests that the tissue tropism of JEV extends to the lungs during intranasal infections. These ideas have not been thoroughly assessed for JEV in particular, as the virus was thought to be transmitted mainly by mosquito vectors. Most interestingly, up to 10^7.0^ RNA copies/g in the lungs were detected in the mice. This efficient replication in the lungs could be explained by local replication at the inoculation site, as we could detect viral RNA in oronasal swabs of the infected mice. A former study showed that the aerosol inoculation of JEV in mice via aerosol-producing machinery fails to detect viral replication in the lungs after 9 dpi [28]. A difference in the viral strain, inoculation route, and dose used could explain the disparity in our results. One previous study found that mice are susceptible to oral challenge, although no molecular data were provided nor was replication in the lungs determined [29]. Not only was shedding detected in the oronasal swabs of the sentinel mice, but infectious viruses were isolated in the lungs of the same animals. Our data demonstrate the transmission of JEV to contact animals from i.n. inoculated animals, which is consistent with previous findings in swine, the natural host [14]. The contact pigs showed viral RNA as high as 10^4.0^ quantities/mL in the sera at 6 and 8 dpi. We showed similar viral RNA loads in the lungs of the contact mice. Contact transmission has also been demonstrated with the Zika virus in guinea pigs in which 100% of the contact animals developed viremia as soon as 1 day post-challenge of greater than 10^5.0^ RNA copies/mL [30]. Normally, humans infected with JEV, similar to mice, do not display apparent viremia or a high viral load in the peripheral organs, except in encephalitic cases where a high viral load is detected in the brain [1]. In contrast, pigs show high viremia following JEV infection [12]. Together, these facts suggest that JEV infection in the peripheral organs is quite different among pigs, mice, and human cases. So far, species differences in viral shedding or transmission remain unclear. Nevertheless, contact transmission could contribute to the spread of JEV during outbreaks within highly concentrated populations in the absence of mosquito vectors, based on our results and former pig studies [12]. 

We show that aerosol transmission is possible in mice, which could greatly impact the study of JEV epidemiology and ecology. Although the vaccine strain SA14-14-2 is less virulent than the SCYA2010201 strain, both the sentinel groups of the animals had viral RNA present in the lungs at 7 dpi. The plaque assays also confirmed the presence of infectious virus in the lungs, albeit to a lesser degree, at 7 dpi, in the sentinel animals. The cage setup completely prevented direct contact of the infected and sentinel animals, further confirming aerosol transmission as the source of infection. Aerosol infection via a nebulizing apparatus has been assessed for JEV in mice, hamsters, guinea pigs, rats, and squirrel monkeys [28]. All these species have the potential to be infected with JEV, with mortality in mice and hamsters. Compared with the intranasal inoculation, the virus can be delivered into the lungs more deeply through an aerosol infection generated by a specific apparatus. No mortality was observed in our study, although a high viral load was detected in the infected mouse lungs and brains. This is probably due to differences in the virus strain and dose and the mouse breeds used in these studies. It is necessary in parallel under the same conditions to compare both intranasal and aerosol infections in JEV tissue tropism and disease progress in future studies. 

Aerosol transmission was previously assessed with respect to the West Nile virus (WNV), duck tembusu, and St. Louis Encephalitis virus (SLEV), all closely related flaviviruses. WNV has been well documented for its potential for aerosol infections in laboratory-acquired infections and in mice [26,31]. In addition to laboratory infections of WNV, the virus can transmit via oronasal infections in a variety of species including mice, alligators, and hamsters [26,32,33]. Considering the similarities between JEV and WNV, we cannot eliminate the potential for alternative routes of transmission for JEV. In 2015, duck tembusu virus was shown to infect ducks via airborne transmission as soon as 4 dpi [34]. Compared to the direct contact ducks, the aerosol-exposed birds showed higher levels of viral RNA, albeit 2 dpi later than the direct contact ducks. The direct contact group showed peak viral titers at 4 dpi of 10^4.0^ RNA copies/mL, while the peak titers for the aerosol-exposed group were 10^5.0^ RNA copies/mL at 6 dpi [30]. Similar to the initial JEV aerosol study, a nebulizing chamber was used at varying infectious doses from 10^2.6^ to 10^7.6^ pfu/mL of SLEV, causing 20–100% mortality, respectively [35]. Taken together, it is reasonable to suggest that JEV is potentially transmissible and infectious via aerosol exposure. 

The viral titers detected in the aerosol-infected group do not reach the level necessary to transmit to mosquitoes. There are potentially two reasons for this. First, mice are not the natural host of JEV, which may limit the viral replication. Second, the low infecting dose may potentially prevent vector-borne transmission from these animals. It is likely that JEV outbreaks are multifactorial, where many forms of disease transmission contribute to the spread and persistence of the disease. In the case of outbreaks with an unknown source, it is speculated that aerosol transmission and persistence may play a role in viral appearance and dissemination. With this in mind, the results of our study provide novel insights into the understanding of JEV epidemiology and might be used to aid in the prediction of future outbreaks. 

## References

[1] Solomon T., Dung N.M., Kneen R., Gainsborough M., Vaughn D.W., Khanh V.T. (2000). Japanese encephalitis. J. Neurol Neurosurg. Psychiatry.

[2] Campbell G.L., Hills S.L., Fischer M., Jacobson J.A., Hoke C.H., Hombach J.M., Marfin A.A., Solomon T., Tsai T.F., Tsu V.D. (2011). Estimated global incidence of Japanese encephalitis: A systematic review. Bull. World Health Organ..

[3] Wang H., Liang G. (2015). Epidemiology of Japanese encephalitis: Past, present, and future prospects. Ther. Clin. Risk Manag..

[4] Weaver S.C., Barrett A.D. (2004). Transmission cycles, host range, evolution and emergence of arboviral disease. Nat. Rev. Microbiol..

[5] Gresser I., Hardy J.L., Hu S.M., Scherer W.F. (1958). Factors influencing transmission of Japanese B encephalitis virus by a colonized strain of Culex tritaeniorhynchus Giles, from infected pigs and chicks to susceptible pigs and birds. Am. J. Trop. Med. Hyg..

[6] Konno J., Endo K., Agatsuma H., Ishida N. (1966). Cyclic outbreaks of Japanese encephalitis among pigs and humans. Am. J. Epidemiol..

[7] Scherer W.F., Moyer J.T., Izumi T. (1959). Immunologic studies of Japanese encephalitis virus in Japan. V. Maternal antibodies, antibody responses and viremia following infection of swine. J. Immunol..

[8] Simpson D.I., Smith C.E., Marshall T.F., Platt G.S., Way H.J., Bowen E.T., Bright W.F., Day J., McMahon D.A., Hill M.N. (1976). Arbovirus infections in Sarawak: The role of the domestic pig. Trans. R. Soc. Trop. Med. Hyg..

[9] Mitchell C.J., Savage H.M., Smith G.C., Flood S.P., Castro L.T., Roppul M. (1993). Japanese encephalitis on Saipan: A survey of suspected mosquito vectors. Am. J. Trop. Med. Hyg..

[10] Takashima I., Hashimoto N., Watanabe T., Rosen L. (1989). Mosquito collection in endemic areas of Japanese encephalitis in Hokkaido, Japan. Nihon Juigaku Zasshi. Jpn. J. Vet. Sci..

[11] Cheng V.C.C., Sridhar S., Wong S.C., Wong S.C.Y., Chan J.F.W., Yip C.C.Y., Chau C.H., Au T.W.K., Hwang Y.Y., Yau C.S.W. (2018). Japanese Encephalitis Virus Transmitted Via Blood Transfusion, Hong Kong, China. Emerg. Infect. Dis..

[12] Lyons A.C., Huang Y.S., Park S.L., Ayers V.B., Hettenbach S.M., Higgs S., McVey D.S., Noronha L., Hsu W.W., Vanlandingham D.L. (2018). Shedding of Japanese Encephalitis Virus in Oral Fluid of Infected Swine. Vector Borne Zoonotic Dis..

[13] Garcia-Nicolas O., Braun R.O., Milona P., Lewandowska M., Dijkman R., Alves M.P., Summerfield A. (2018). Targeting of the Nasal Mucosa by Japanese Encephalitis Virus for Non-Vector-Borne Transmission. J. Virol..

[14] Ricklin M.E., Garcia-Nicolas O., Brechbuhl D., Python S., Zumkehr B., Nougairede A., Charrel R.N., Posthaus H., Oevermann A., Summerfield A. (2016). Vector-free transmission and persistence of Japanese encephalitis virus in pigs. Nat. Commun..

[15] Yuan L., Wu R., Liu H., Wen X., Huang X., Wen Y., Ma X., Yan Q., Huang Y., Zhao Q. (2016). Tissue tropism and molecular characterization of a Japanese encephalitis virus strain isolated from pigs in southwest China. Virus Res..

[16] Yun S.I., Song B.H., Polejaeva I.A., Davies C.J., White K.L., Lee Y.M. (2016). Comparison of the live-attenuated Japanese encephalitis vaccine SA14-14-2 strain with its pre-attenuated virulent parent SA14 strain: Similarities and differences in vitro and in vivo. J. Gen. Virol..

[17] Yu Y. (2010). Phenotypic and genotypic characteristics of Japanese encephalitis attenuated live vaccine virus SA14-14-2 and their stabilities. Vaccine.

[18] Zhao Z., Wu G., Zhu X., Yan X., Dou Y., Li J., Zhu H., Zhang Q., Cai X. (2012). RNA interference targeting virion core protein ORF095 inhibits Goatpox virus replication in Vero cells. Virol. J..

[19] Liu H., Wu R., Yuan L., Tian G., Huang X., Wen Y., Ma X., Huang Y., Yan Q., Zhao Q. (2017). Introducing a cleavable signal peptide enhances the packaging efficiency of lentiviral vectors pseudotyped with Japanese encephalitis virus envelope proteins. Virus Res..

[20] Yuan L., Wu R., Liu H., Wen X., Huang X., Wen Y., Ma X., Yan Q., Huang Y., Zhao Q. (2016). The NS3 and NS4A genes as the targets of RNA interference inhibit replication of Japanese encephalitis virus in vitro and in vivo. Gene.

[21] Fan Y.C., Chen J.M., Chiu H.C., Chen Y.Y., Lin J.W., Shih C.C., Chen C.M., Chang C.C., Chang G.J., Chiou S.S. (2012). Partially neutralizing potency against emerging genotype I virus among children received formalin-inactivated Japanese encephalitis virus vaccine. PLoS Negl. Trop. Dis..

[22] Liu H., Wu R., Liu K., Yuan L., Huang X., Wen Y., Ma X., Yan Q., Zhao Q., Wen X. (2016). Enhanced immune responses against Japanese encephalitis virus using recombinant adenoviruses coexpressing Japanese encephalitis virus envelope and porcine interleukin-6 proteins in mice. Virus Res..

[23] German A.C., Myint K.S., Mai N.T., Pomeroy I., Phu N.H., Tzartos J., Winter P., Collett J., Farrar J., Barrett A. (2006). A preliminary neuropathological study of Japanese encephalitis in humans and a mouse model. Trans. R. Soc. Trop. Med. Hyg..

[24] Saxena V., Mathur A., Krishnani N., Dhole T.N. (2008). Kinetics of cytokine profile during intraperitoneal inoculation of Japanese encephalitis virus in BALB/c mice model. Microbes Infect..

[25] Aliota M.T., Caine E.A., Walker E.C., Larkin K.E., Camacho E., Osorio J.E. (2016). Characterization of Lethal Zika Virus Infection in AG129 Mice. PLoS Negl. Trop. Dis..

[26] Nir Y., Beemer A., Goldwasser R.A. (1965). West Nile Virus infection in mice following exposure to a viral aerosol. Br. J. Exp. Pathol..

[27] Sun E., Zhao J., Tao Y., Xu Q., Qin Y., Wang W., Wei P., Wu D. (2013). Antibodies generated by immunization with the NS1 protein of West Nile virus confer partial protection against lethal Japanese encephalitis virus challenge. Vet. Microbiol..

[28] Larson E.W., Dominik J.W., Slone T.W. (1980). Aerosol stability and respiratory infectivity of japanese B encephalitis virus. Infect. Immun..

[29] Ramakrishna C., Desai A., Shankar S.K., Chandramuki A., Ravi V. (1999). Oral immunisation of mice with live Japanese encephalitis virus induces a protective immune response. Vaccine.

[30] Deng Y.Q., Zhang N.N., Li X.F., Wang Y.Q., Tian M., Qiu Y.F., Fan J.W., Hao J.N., Huang X.Y., Dong H.L. (2017). Intranasal infection and contact transmission of Zika virus in guinea pigs. Nat. Commun..

[31] Nir Y.D. (1959). Airborne West Nile virus infection. Am. J. Trop. Med. Hyg..

[32] Klenk K., Snow J., Morgan K., Bowen R., Stephens M., Foster F., Gordy P., Beckett S., Komar N., Gubler D. (2004). Alligators as West Nile virus amplifiers. Emerg. Infect. Dis..

[33] Reagan R.L., Yancey F.S., Chang S.C., Brueckner A.L. (1956). Transmission of West Nile (B956 strain) and Semliki Forest virus (MBB26146-M-404744-958 strain) to suckling hamsters during lactation. J. Immunol..

[34] Li X., Shi Y., Liu Q., Wang Y., Li G., Teng Q., Zhang Y., Liu S., Li Z. (2015). Airborne Transmission of a Novel Tembusu Virus in Ducks. J. Clin. Microbiol..

[35] Phillpotts R.J., Brooks T.J., Cox C.S. (1997). A simple device for the exposure of animals to infectious microorganisms by the airborne route. Epidemiol. Infect..

